# A Novel Method for Fracture Blister Management Using Circumferential Negative Pressure Wound Therapy with Instillation and Dwell

**DOI:** 10.7759/cureus.3509

**Published:** 2018-10-29

**Authors:** Ian G Hasegawa, John P Livingstone, Patrick Murray

**Affiliations:** 1 Orthopedic Surgery, University of Hawaii, John A. Burns School of Medicine, Honolulu, USA; 2 Orthopedic Surgery, Queen's Medical Center, Honolulu, USA

**Keywords:** fracture blisters, negative pressure wound therapy, wound, trauma, soft tissue, negative pressure wound therapy with instillation and dwell

## Abstract

High- and low-energy fractures can result in nearby skin blistering. These so-called “fracture blisters” can be troublesome in the face of surgery and currently no uniform consensus regarding their management exists. Preoperatively, we used circumferential negative pressure wound therapy with sterile saline instillation (NPWT-id) to treat two patients with closed fractures who had developed significant skin blistering. This technique resulted in near complete re-epithelialization of the decompressed blister beds within one week. Furthermore, no excessive surgical delay or alteration in surgical approach was necessary, and both patients healed successfully without post-operative wound complications. Thus, circumferential NPWT-id may be a worthwhile treatment option for fracture blisters.

## Introduction

Significant soft tissue swelling and the development of skin blisters, so-called “fracture blisters”, are a common and often unavoidable consequence of high- and low-energy fractures. Fracture blisters can be troublesome to the orthopedic surgeon as they have been associated with a delay in time to surgery, suboptimal surgical approaches, and wound complications [1-3]. Currently no uniform consensus regarding the optimal management of fracture blisters exists. While most agree that fracture blisters should not be directly operated through, recommendations have varied. These include observation and surgical delay, to deroofment and topical antibiotics [1,2].

Recently much attention has surrounded the successful use of negative pressure wound therapy (NPWT) for the management of traumatic orthopedic wounds and surgical incisions. Two proposed mechanisms include reduction in wound edema and improvement in tissue perfusion [4]. Varela et al. described a close link between posttraumatic edema, local venous stasis and thrombosis, and the development of epidermal necrosis and subsequent skin blistering [3]. With this in mind, NPWT appears to be a plausible method to treat, and possibly even prevent the development of fracture blisters.

To our knowledge, no studies currently exist regarding the treatment of fracture blisters with NPWT. We present two case reports in which fracture blisters arising from an ankle and tibial plateau fracture were treated successfully with preoperative negative pressure wound therapy with instillation and dwell (NPWT-id). Additionally, we provide a detailed description of our NPWT-id application technique.

## Case presentation

Case 1

History

**Figure 1:**
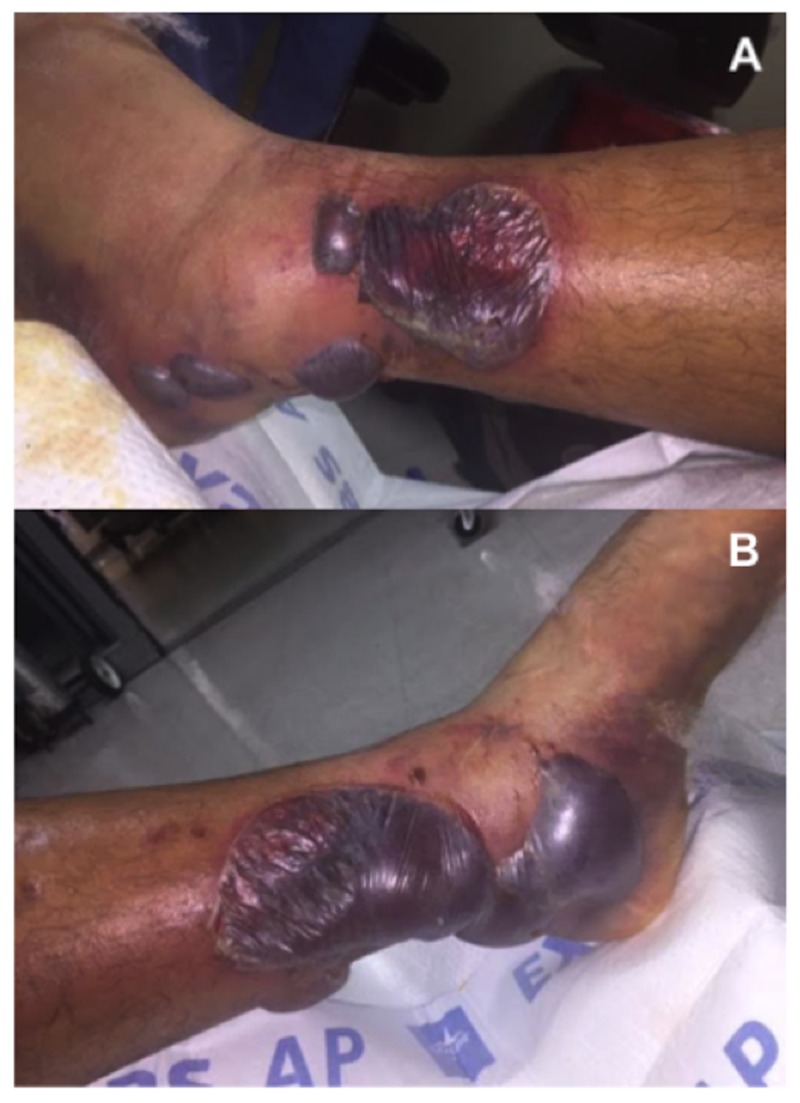
Hemorrhagic fracture blisters. (A) Lateral ankle, (B) medial ankle.

A 54-year-old male, with a history of non-insulin dependent diabetes mellitus and polysubstance abuse, presented to the emergency department with an isolated closed left bimalleolar ankle fracture after jumping down from a fence. He was initially diagnosed three days prior at an outside hospital where he was placed in a custom molded fiberglass splint. Upon removal of the splint, multiple hemorrhagic skin blisters were found to the medial and lateral ankle (Figure 1). The patient denied any interval trauma. Additionally, although he remained in the splint since its initial application, he noted noncompliance with non-weight bearing precautions.

Physical Exam and Diagnosis

**Figure 2:**
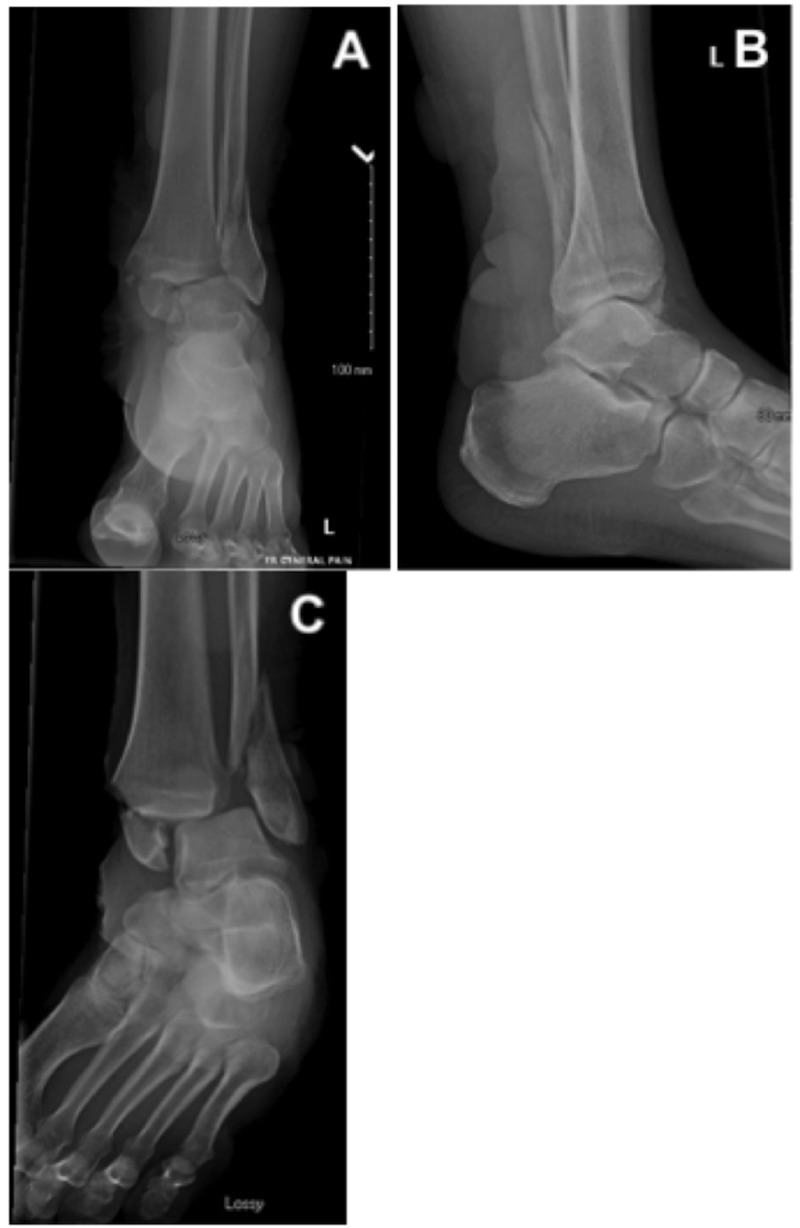
Injury radiographs. (A) Anterior-posterior, (B) lateral, and (C) mortise ankle radiographs demonstrating a bimalleolar ankle fracture involving the medial malleolus and distal fibula with lateral talar subluxation.

Significant swelling, ecchymosis, and tenderness of the left ankle were noted. There were large intact hemorrhagic blisters both medially and laterally. An overlying blister did prevent assessment of the posterior tibial pulse. However, a strong dorsalis pedis pulse and brisk capillary refill of all digits were noted. No deficits in distal motor or sensation were found. Ankle radiographs demonstrated a displaced medial malleolus and distal fibula fracture with lateral talar subluxation (Figure 2). Baseline labs, including a complete blood count, basic metabolic panel, erythrocyte sedimentation rate (ESR) and c-reactive protein (CRP), were ordered and found to be within normal ranges.

NPWT-id Technique

**Figure 3:**
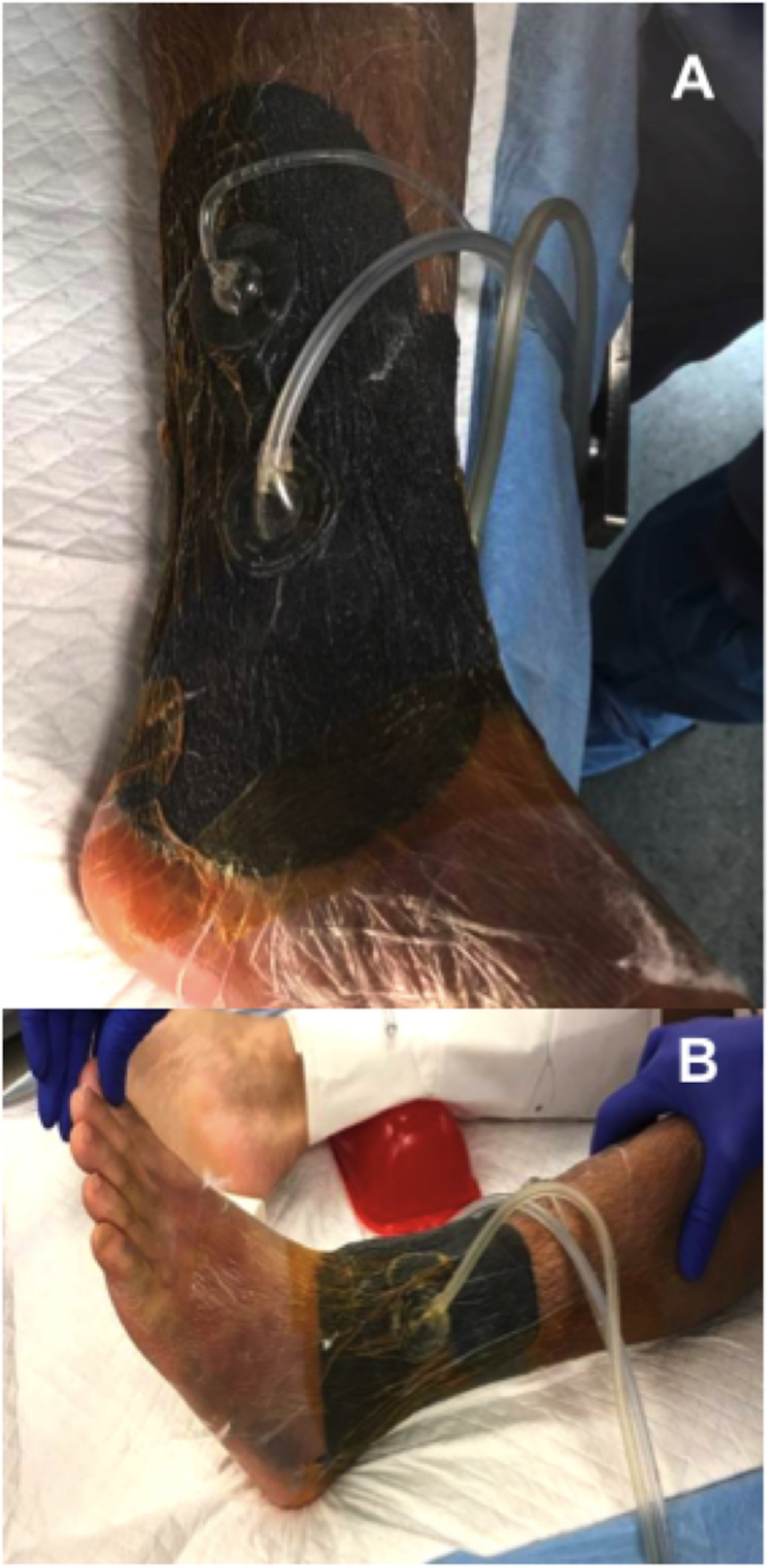
Preoperative circumferential NPWT-id. (A) Medial ankle, (B) lateral ankle. NPWT-id: Negative pressure wound therapy with instillation and dwell

An intraarticular hematoma block was provided via the anteromedial ankle. Closed reduction was then performed under mini c-arm fluoroscopic guidance. A circumferential VeraFlo (Acelity, San Antonio, TX, USA) wound vac was then applied prior to splint application (Figure 3). During wound vac application, tibiotalar reduction was maintained by holding the ankle in a dorsiflexed and supinated position. Adequate reduction was important to prevent excessive skin tension that would result from fracture displacement. We then decompressed all fracture blisters with the tip of a scalpel blade. The overlying epidermis was left in place. Next, the ankle was lined circumferentially with one-inch adhesive strips at the most proximal and distal fracture blisters margins. VeraFlo sponge was then customized to fit within this lined region. Once fitted, the sponge was sealed using adhesive drapes. Prior to sealing the sponge, adhesive drape sheets were quartered. When sealing the sponge, care was taken to lay the adhesive drapes with as little tension on the skin as possible. Instillation settings were as follows: normal saline at 30 ml soak volume, one-minute soak time, at two-hour intervals. Suction was set at negative one hundred, 25 mmHg. Finally a “U splint” with bulky cast padding was applied.

Hospital Course

The patient was placed on strict non-weight bearing precautions to the left lower extremity. He was instructed to elevate the ankle while in bed. His diabetes was managed by our institutions’ hospitalist service. Additionally, the patient was placed on moderate dose lovenox to minimize the risk of deep venous thrombosis (DVT) formation. Anticoagulation was subsequently discontinued 24 hours prior to surgery. Daily assessment of swelling was started on hospital day three. While the splint was removed during these checks, at no time was the VeraFlo device removed or exchanged.

Operative Course

**Figure 4:**
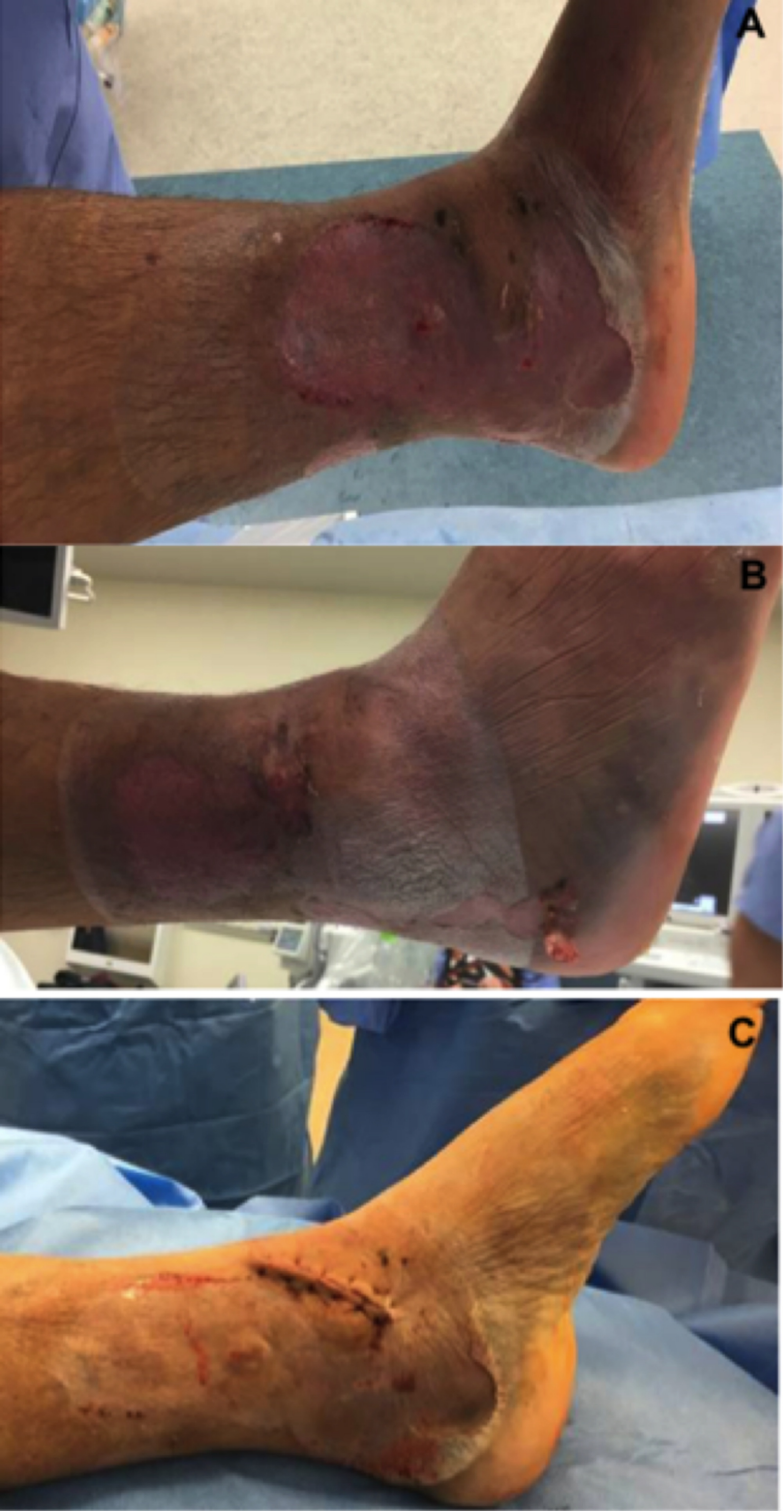
Soft tissue assessment of ankle. (A) Medial and (B) lateral ankle following one week of NPWT-id. Near complete reepithelialization of the blister bed is demonstrated. (C) Primary closure of anteromedial ankle incision that was performed directly through medial blister bed. NPWT-id: Negative pressure wound therapy with instillation and dwell

**Figure 5:**
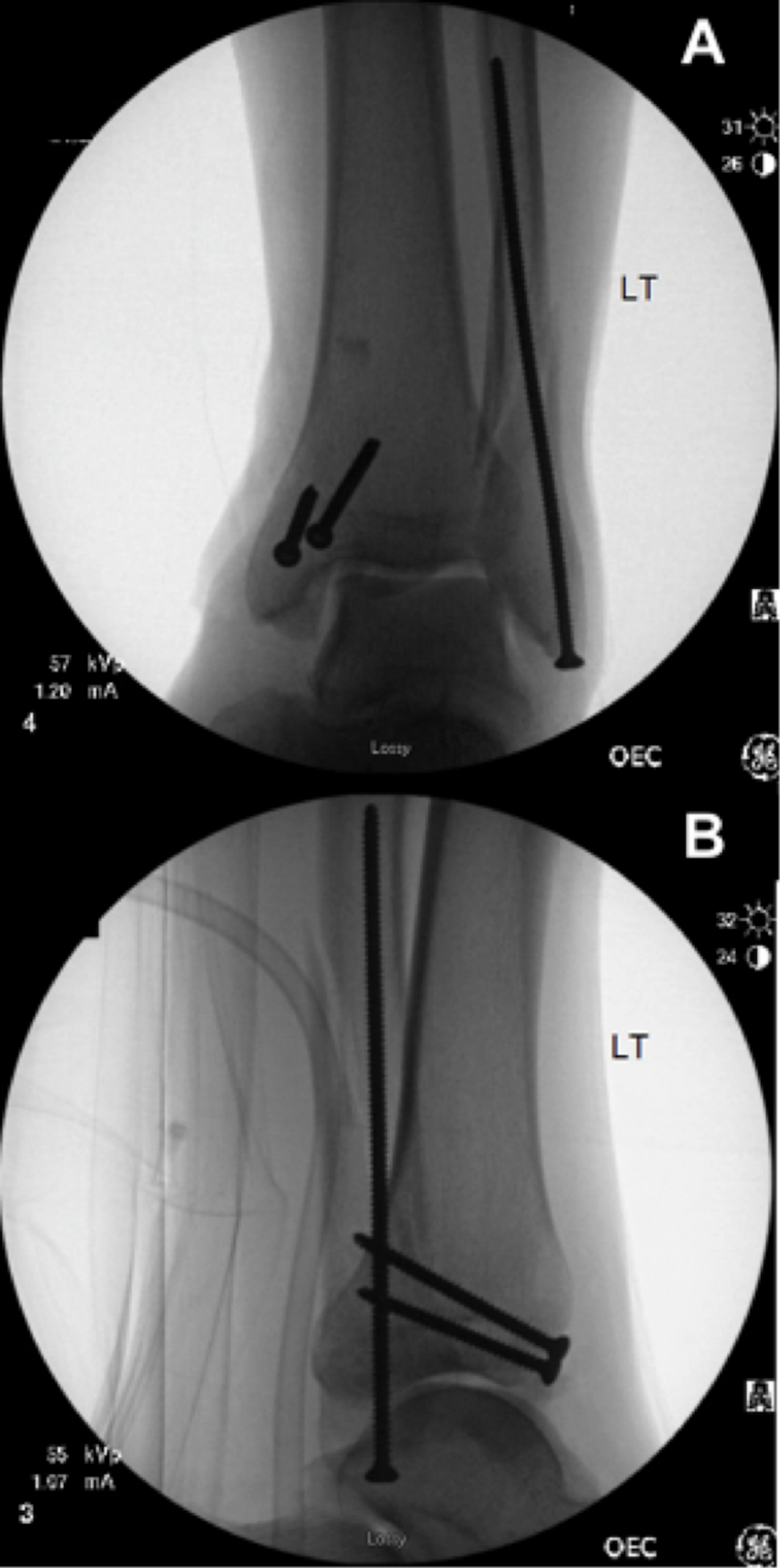
Post-fixation radiographs. (A) Intraoperative anterior-posterior and (B) lateral fluoroscopic radiographs demonstrating percutaneous screw fixation of the medial and lateral malleoli.

**Figure 6:**
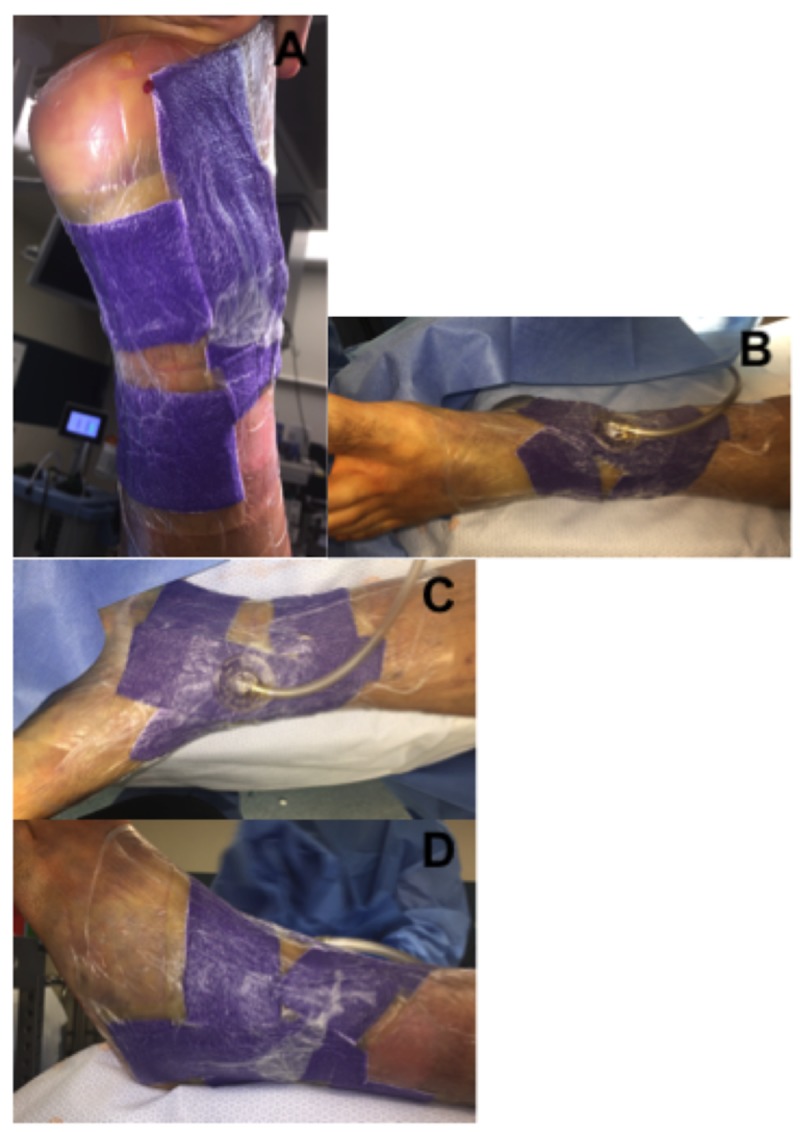
Postoperative circumferential PREVENA Plus wound vac. A circumferential PREVENA Plus wound vac was applied prior to splint application. (A) Posterior, (B) anterior, (C) medial, and (D) lateral views.

On hospital day seven, the patient underwent left ankle open reduction internal fixation. Upon removal of the VeraFlo device, the epidermal skin layer of all decompressed fracture blisters had completely necrosed. This tissue was easily removed with moist gauze. Removal of this necrotic layer revealed near complete reepithelialization of the underlying blister bed (Figure 4). Additionally, while there was mild maceration of the intact skin surrounding the fracture blisters, the skin appeared relatively uninjured. Given the healthy skin appearance and significant reduction in soft tissue swelling, we decided to proceed with open reduction internal fixation using small incisions at the anteromedial and lateral ankle (Figure 5). Post-fixation, all surgical incision sites were closed primarily using 3-0 nylon sutures. A circumferential PREVENA Plus (Acelity, San Antonio, TX, USA) wound vac set at negative one hundred, 25 mmHg continuous suction and U splint were then applied prior to awakening (Figure 6).

Results

**Figure 7:**
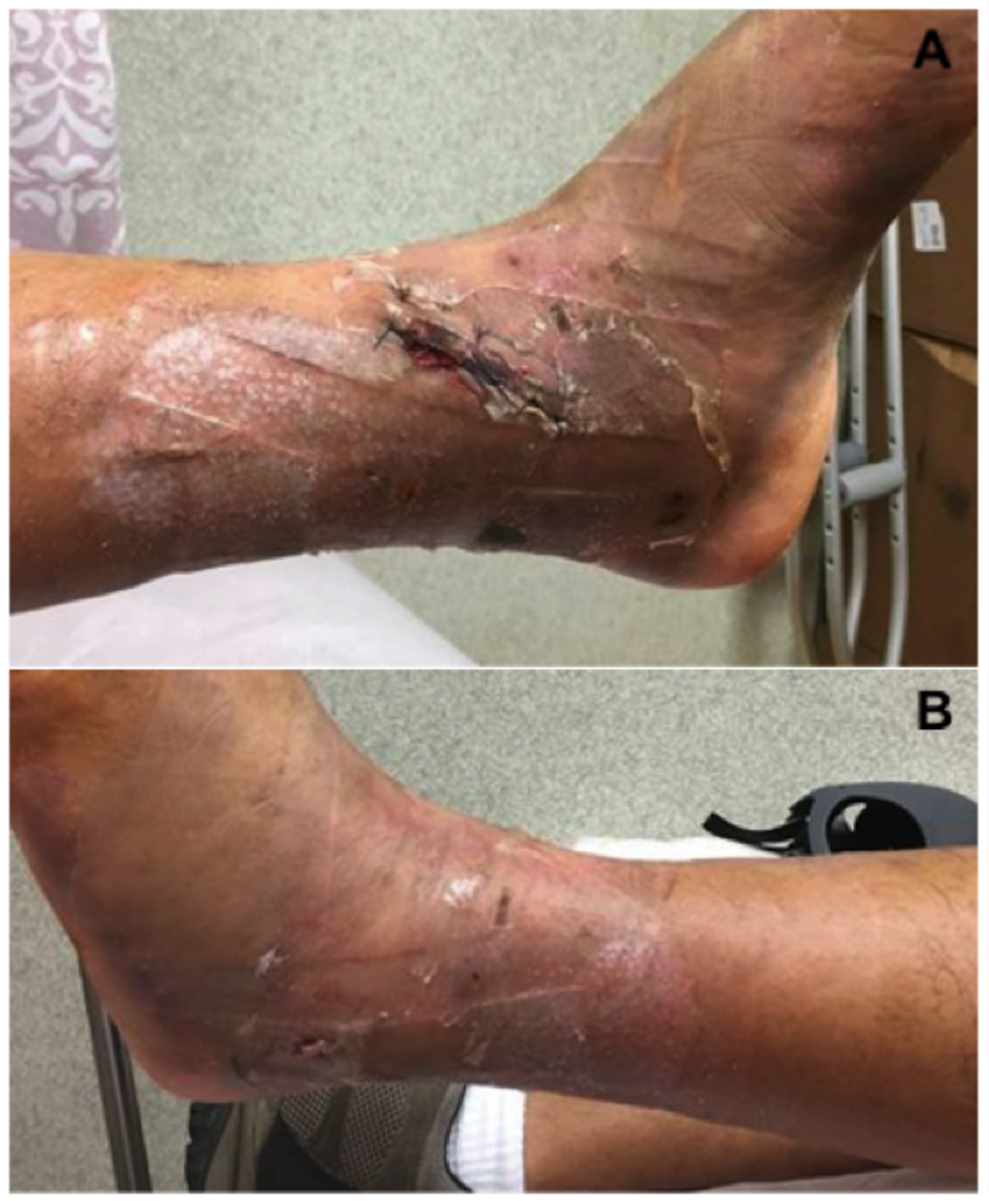
Left ankle surgical incisions at one-week follow-up.

The patient’s postoperative hospital course was uncomplicated. He did receive 24 hours of intravenous antibiotics and was started on moderate dose lovenox for deep venous thrombosis prophylaxis on postoperative day one. He was subsequently discharged on postoperative day two. First outpatient follow-up occurred one week from the day of surgery. He remained in the circumferential PREVENA Plus wound vac and splint up until this point. Upon removal of the splint and wound vac, complete reepithelialization was observed at all fracture blister beds. While there was mild superficial skin edge necrosis at the anteromedial incision site, both the anteromedial and lateral incision sites were well healing and non-draining (Figure 7). Sutures were removed at this visit and the patient was placed in a fracture boot for four additional weeks. At the six-week follow-up all surgical sites demonstrated complete wound healing. No further wound complications were noted.

Case 2

History and Physical Exam

**Figure 8:**
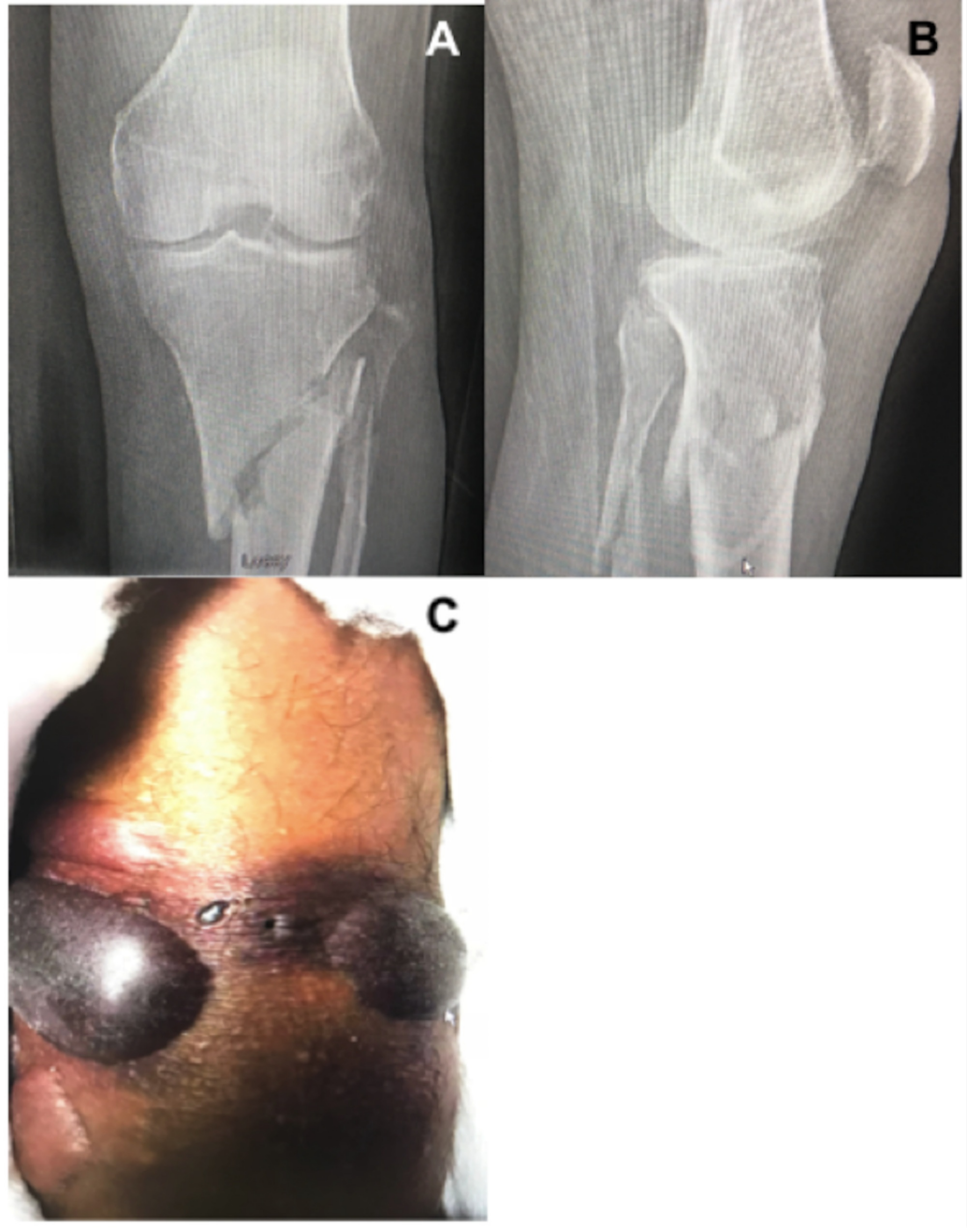
Tibial plateau injury radiographs and fracture blisters. (A) Anterior-posterior (AP) and (B) lateral radiographs demonstrating a Schatzker 6 tibial plateau fracture. (C) Large hemorrhagic fracture blisters to the anteromedial and lateral proximal tibia.

A 62-year-old male, medical history significant for non-insulin dependent diabetes mellitus, who presented with an isolated closed tibial plateau fracture, Schatzker type 6, suffered from blunt force trauma (Figure 8). At the time of presentation significant swelling of the proximal tibia with two large hemorrhagic fracture blisters anteriorly were noted (Figure 8). Despite the high degree of swelling there was no clinical evidence of compartment syndrome and the patient remained neurovascularly intact.

NPWT Technique

**Figure 9:**
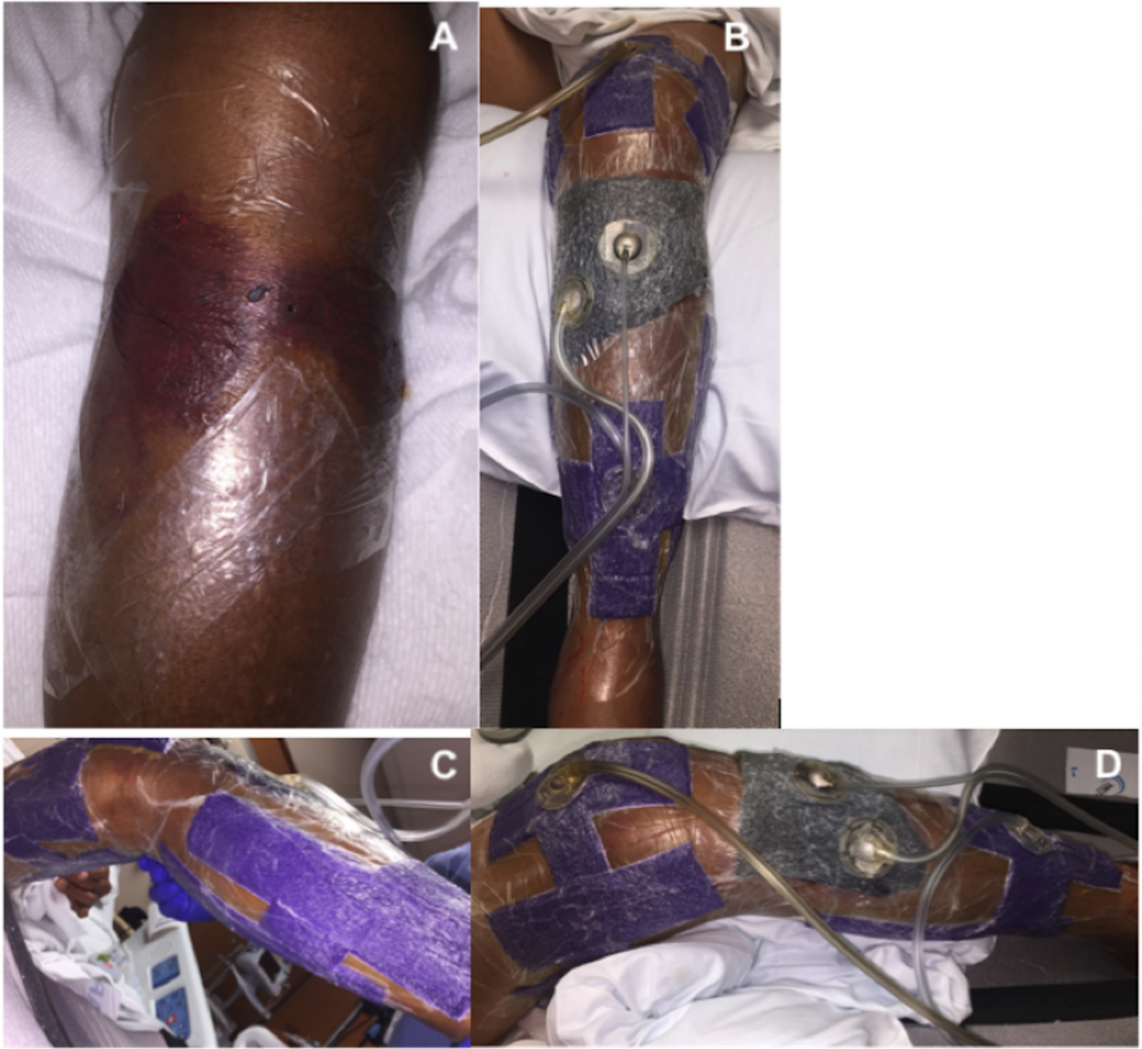
Preoperative circumferential NPWT-id of tibial plateau fracture. (A) Decompressed fracture blisters lined with advanced drapes at blister margins. (B-D) Final application of circumferential wound vac. VeraFlo sponge was applied over the fracture blister region while PREVENA Plus sponge was used to circumferentially span the remaining proximal tibia and distal femur. NPWT-id: Negative pressure wound therapy with instillation and dwell

Similarly, surgery was delayed due to the degree of swelling and skin compromise. A circumferential wound vac consisting of both VeraFlo and PREVENA Plus was placed (Figure 9). Fracture blisters were first decompressed without removal of the overlying epidermis. Fracture blister edges were then lined with one-inch strips of adhesive drapes. Veraflo sponge was customized to fill the lined area and subsequently sealed. Normal saline was instilled at a volume of 20 ml with one-minute soak times, at two-hour intervals. Suction was set at negative one hundred, 25 mmHg. Customized strips of PREVENA Plus foam were then applied circumferentially to the remaining tibia and distal femur as pictured in Figure 9. Attention was made to avoid the popliteal fossa with both the foam and adhesive drapes. Adhesive drapes were placed without tensioning the skin. Continuous suction for the PREVENA Plus wound vac was also set at negative one hundred, 25 mmHg. A knee immobilizer was then applied and the patient was placed on non-weight bearing precautions. Swelling was monitored daily, however, the wound vac was left in place.

Operative Course

**Figure 10:**
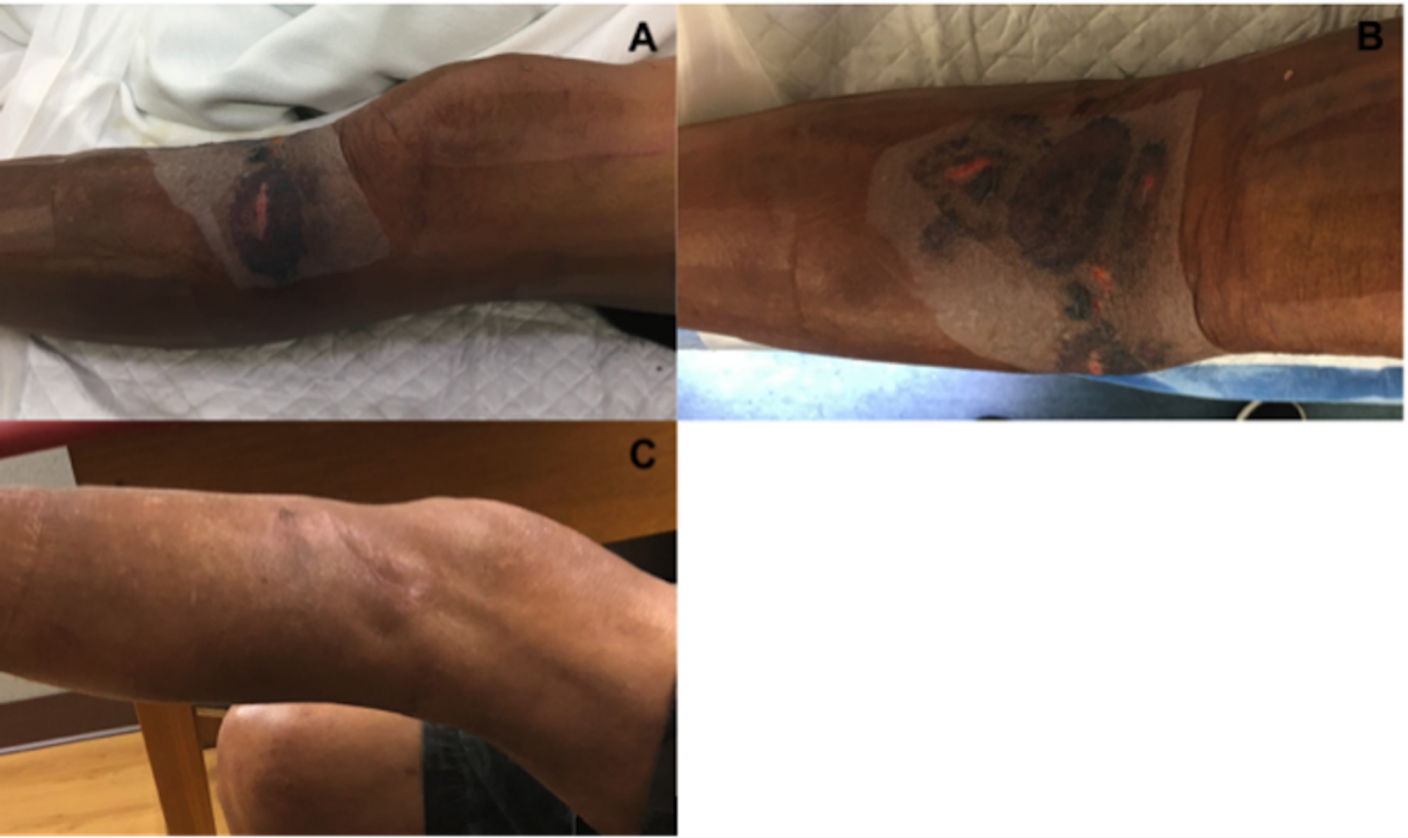
Soft tissue assessment prior to surgery and at six weeks postoperatively. (A,B) Decompressed fracture blisters following one-week of NPWT-id. (C) Completely healed anterolateral surgical incision at six-week postoperative follow-up. NPWT-id: Negative pressure wound therapy with instillation and dwell

On hospital day seven, the patient was scheduled for open reduction internal fixation. Once under general anesthesia the wound vac was removed. There was a significant decrease in the degree of swelling to the proximal tibia. The overlying epidermal layer of the fracture blisters had completely necrosed (Figure 10). Upon removal of this layer with moist gauze, we again observed near complete reepithelialization of the blister bed. Additionally, we observed mild maceration to the intact skin that was covered with VeraFlo sponge. Given the healthy appearance of the skin and significant reduction in soft tissue swelling we proceeded with open reduction internal fixation. A single skin incision utilizing a standard anterolateral approach was utilized without alteration (Figure 10). Following reduction and fixation with a lateral plate and a combination of locking and nonlocking screws, the wound was closed primarily with deep 2-0 vicryl and superficial 2-0 nylon sutures. A circumferential PREVENA Plus wound vac was applied to the proximal tibia and distal femur as previously described. Continuous suction was set at negative one hundred, 25 mmHg.

Results

The patient completed 24 hours of post-operative intravenous antibiotics and moderate dose lovenox DVT prophylaxis was started on postoperative day one. He was subsequently discharged from the hospital on postoperative day two. Wound checks were performed at one and six weeks following hospital discharge (Figure 10). His wounds went on to heal completely without complications.

## Discussion

To our knowledge, this is the first written report on the utilization of NPWT-id for the management of fracture blisters. In both cases, our most important findings were the near complete reepithelialization of all fracture blister beds following one week of NPWT-id and the absence of post-surgical wound complications. Additionally, no extended surgical delay or alteration in surgical approach was necessary.

Currently, little is known about the optimal handling of skin blisters arising in the setting of acute fractures. Multiple studies suggest surgical delay and alternative surgical approaches to reduce the risk of wound complications [1-3]. Despite these adjustments wound complications still occur at high rates [1-3]. Strauss et al. reported a 13% wound complication rate in their study of 45 patients with lower extremity fracture blisters [1]. In this study, all patients received blister deroofment followed by twice daily silver sulfadiazine (Silvadene) application to the blister bed. Two major wound complications were noted in patients with a history of non-insulin dependent diabetes mellitus. This included a deep infection requiring revision surgery and ultimate ankle fusion, and a persistent medial wound breakdown requiring hardware removal. It is also important to note that their overall average surgical delay was seven days. Furthermore, a significant difference in surgical delay was found between ankle and tibial plateau fractures, which averaged six and 11 days each, respectively (p < 0.05).

In a prospective analysis, Giordano and Koval compared various treatment modalities including blister aspiration, deroofment plus silvadene application, and leaving the blister intact [2]. No significant difference in blister healing was found between the different modalities. Eighty-seven percent had reepithelialized their blister beds at an average of three months while 13% suffered wound complications requiring subsequent skin grafting in all but one. It is important to note that all wound complications were associated with a blood-filled blister type. Additionally, in two of these cases the surgical incision was made directly through a blood-filled blister bed. Both of these cases experienced wound complications requiring split thickness skin grafting. No wound complications were noted when incisions were made through clear fluid-filled blister beds. The authors’ final recommendation was to leave all blisters untreated unless spontaneous rupture occurs.

Varela et al. also found no significant difference in fracture blister healing between those treated with dry dressings, silvadene application, or whirlpool debridements plus silvadene application [3]. In this study all blisters were left to rupture spontaneously. More importantly, these authors performed a histologic and microbial analysis on 15 of the fracture blisters. Their analysis confirmed that fracture blisters are subepidermal vesicles filled with sterile transudate. Furthermore, their microbial analysis indicated rapid colonization of the blister bed with opportunistic skin flora, namely S. epidermidis and S. aureus, shortly following blister rupture.

In contrast to the findings of Strauss et al. and Giordano and Koval, we did not observe any post-operative wound complications despite operating through blood-filled blister beds in patients with a history of non-insulin dependent diabetes [1,2]. It is possible that this may be due to our small sample size and shorter follow-up period. However, given the near complete reepithelialized blister beds and limited soft tissue swelling at the time of surgery, we were likely operating through soft tissue that had adequately recovered from the initial trauma. Additionally, the absence of post-operative wound complications, including further blister development, may be attributed to our post-surgical application of a circumferential PREVENA Plus wound vac.

Multiple studies demonstrate increased tissue perfusion, decreased wound edema, and stimulation of granulation tissue following NPWT. When comparing traditional NPWT with NPWT-id, Omar et al. found faster wound healing times when NPWT was combined with sterile saline instillation [5]. Together, these factors likely accounted for our faster reepithelialization time, significant reduction in soft tissue swelling, and consequently shorter delay to surgery. It is also possible that our circumferential application of wound vacs may have enhanced these responses given the larger regional surface area covered. However, this has not been tested in the literature yet and as such remains theoretical only.

As suggested by Varela et al., the high rate of wound complications previously noted may in part be due to the rapid colonization of blister beds with staph species [3]. In our study, we demonstrated no wound complications at any time point despite the intentional rupture of all fracture blisters. We believe this to be due to our use of sterile saline instillation. It is well established that NPWT without instillation does not play a large role in reducing bacterial load in wounds. However, several recent studies demonstrated a significant reduction in bioburden in chronic wounds after seven days of NPWT-id [6,7]. While these studies instilled an antimicrobial solution, we chose to instill sterile saline given the relatively clean appearance of the skin prior to wound vac application, as well as the well-established efficacy of normal saline irrigation on traumatic and chronic wounds. Still, further research is required to determine the true efficacy of NPWT with saline instillation on bioburden reduction.

It is also important to point out that in both cases, VeraFlo sponge was applied directly over intact skin that surrounded the fracture blisters. Specifically, in case one, this represented a relatively large area anteriorly and over the lateral malleolus (Figures 1, 3 ). Interestingly, this resulted in very mild skin maceration (Figures 4, 10). Thus, while we do not believe that the use of NPWT-id over intact skin was necessary to achieve our results, we do believe that one week of NPWT-id with short dwell times can be used safely over intact skin. It is possible that our one minute saline soak times prevented further skin injury that is typically expected when traditional wound vac sponge is applied directly to intact skin with continuous suction. However, to our knowledge this has not yet been tested.

The major limitations of this study include our small sample size, short term follow-up, and lack of a comparative group. As such, future research should include a larger sample size, longer follow-up, and comparison trials. For instance, comparing NPWT versus NPWT-id versus PREVENA plus, as well as NPWT-id with sterile saline versus an antimicrobial solution to more accurately determine the efficacy of NPWT with sterile saline instillation on fracture blister management.

## Conclusions

We demonstrated the successful management of fracture blisters treated with decompression followed by circumferential NPWT combined with sterile saline instillation. Our findings suggest that this technique may lead to faster reepithelialization times, shorter time to surgery, reduced need for alternative surgical approaches, and lower rates of post-operative wound complications. While further research is required to determine the true efficacy of NPWT-id on fracture blister handling, this technique appears promising and as such should be included in one’s armamentarium when dealing with fracture blister.
